# The Importance of the Ionic Product for Water to Understand the Physiology of the Acid-Base Balance in Humans

**DOI:** 10.1155/2014/695281

**Published:** 2014-04-30

**Authors:** María M. Adeva-Andany, Natalia Carneiro-Freire, Cristóbal Donapetry-García, Eva Rañal-Muíño, Yosua López-Pereiro

**Affiliations:** Hospital General Juan Cardona, C/ Pardo Bazán s/n, Ferrol, 15406 La Coruña, Spain

## Abstract

Human plasma is an aqueous solution that has to abide by chemical rules such as the principle of electrical neutrality and the constancy of the ionic product for water. These rules define the acid-base balance in the human body. According to the electroneutrality principle, plasma has to be electrically neutral and the sum of its cations equals the sum of its anions. In addition, the ionic product for water has to be constant. Therefore, the plasma concentration of hydrogen ions depends on the plasma ionic composition. Variations in the concentration of plasma ions that alter the relative proportion of anions and cations predictably lead to a change in the plasma concentration of hydrogen ions by driving adaptive adjustments in water ionization that allow plasma electroneutrality while maintaining constant the ionic product for water. The accumulation of plasma anions out of proportion of cations induces an electrical imbalance compensated by a fall of hydroxide ions that brings about a rise in hydrogen ions (acidosis). By contrast, the deficiency of chloride relative to sodium generates plasma alkalosis by increasing hydroxide ions. The adjustment of plasma bicarbonate concentration to these changes is an important compensatory mechanism that protects plasma pH from severe deviations.

## 1. Introduction

Although invaluable contributions to the understanding of the acid-base balance in humans have been made, the physiological mechanisms that justify variations of plasma pH in different conditions are unclear and therefore the therapy of the acid-base disorders has remained elusive.

The constancy of the ionic product for water is an essential missing piece of information that may contribute to elucidate the pathophysiology of the acid-base balance in humans.

Plasma and urine are aqueous solutions. As such, they have to comply with chemical rules that apply to aqueous solutions, including the principle of electrical neutrality and the constancy of the ionic product for water. The state of ionization of plasma water varies according to the plasma ionic composition to maintain these chemical rules.

## 2. The Principle of Electrical Neutrality

Electrolytes are molecules that dissociate in water forming cations (positively charged ions) and anions (negatively charged ions). In humans, plasma cations include hydrogen ion (H^+^), sodium, potassium, calcium, magnesium, and ammonium. Sodium ion contributes quantitatively the principal positive charge to plasma, with a concentration of approximately 140 mmol/L (mM) (0.140 M), while normal plasma concentration of hydrogen ions is 0.00000004 mol/L (M) or 40 nanoM (nM). Plasma anions include hydroxide ion (OH^−^, also termed hydroxyl ion), chloride, bicarbonate (HCO_3_^−^), albumin, phosphate, lactate, *β*-hydroxybutyrate, acetoacetate, sulphate, urate, and other organic anions such as pyruvate and propionate. The most abundant anion in human plasma is chloride with a concentration of approximately 100 mM. Plasma bicarbonate level is approximately 25 mM and therefore also contributes significantly to the plasma negative charge in quantitative terms.

In order to preserve normal plasma ionic composition, the kidneys produce urine with different concentration of ions depending on body requirements. Urine cations include sodium, potassium, calcium, magnesium, ammonium, and hydrogen ions. Urine anions include chloride, bicarbonate, phosphate, sulphate, citrate, oxalate, and hydroxide ions.

According to the electroneutrality principle, the sum of all positively charged ions (cations) must be equal to the sum of all negatively charged ions (anions) in aqueous solutions. Therefore, plasma and urine are electrically neutral and the sum of their anions (negative electrical charge) equals the sum of their cations (positive electrical charge) [1–4].

## 3. The Constancy of the Ionic Product for Water

The ionic product for water is a crucial piece to understand acid-base balance physiology and to evaluate acid-base disorders. In water, some water molecules accept a hydrogen ion from a second water molecule, forming a hydronium ion (H_3_O^+^, also called hydroxonium ion or oxonium ion) and a hydroxide ion (OH^−^). Once generated, both ions react to produce water again, according to the following equilibrium reaction:

(1) $$\begin{array}{c} {\text{H}}_{2}\text{O}+{\text{H}}_{2}\text{O}⟷{\text{H}}_{3}{\text{O}}^{+}+{\text{OH}}^{-} \end{array}$$

By convenience, the hydronium ion (H_3_O^+^) is usually identified as hydrogen ion (H^+^) and the equilibrium reaction can be rewritten as

(2) $$\begin{array}{c} {\text{H}}_{2}\text{O}+{\text{H}}_{2}\text{O}⟷{\text{H}}^{+}+{\text{OH}}^{-} \end{array}$$

The law of mass action can be applied, defining the equilibrium constant for the reversible ionization of water (*K*_eq_):

(3) $$\begin{array}{c} K_{\text{eq}}=\frac{\left[{\text{H}}^{+}\right]\left[\text{O}{\text{H}}^{-}\right]}{\left[{\text{H}}_{2}\text{O}\right]}. \end{array}$$

The concentration of each moiety is expressed in moles per liter (M).

The degree of ionization of water is very low and the number of water molecules dissociated into ions is minuscule. At any given time, the amount of hydronium ions and hydroxide ions present in water is extremely small and consequently the concentration of undissociated water molecules ([H_2_O]) is virtually unchanged by this minute ionization and may be considered a constant.

(4) $$\begin{array}{c} K_{\text{eq}}\times \left[{\text{H}}_{2}\text{O}\right]=\left[{\text{H}}^{+}\right]\left[\text{O}{\text{H}}^{-}\right]. \end{array}$$

The product *K*_eq_ × [H_2_O] is a constant termed the ionic product for water (*K*_*w*_).

Therefore, the ionic product for water (*K*_*w*_) is

(5) $$\begin{array}{c} K_{w}=\left[{\text{H}}^{+}\right]\left[\text{O}{\text{H}}^{-}\right]. \end{array}$$

In pure water, the concentration of water is 55.5 M and the value for the equilibrium constant, *K*_eq_, determined by electrical conductivity measurements, is 1.8 × 10^−16^ M at 25°C (298 K) of temperature.

Substituting these values in the equilibrium constant expression,

(6) $$\begin{array}{c} K_{w}=\left(1.8\times 10^{-16}\right)\times 55.5=99.9\times 10^{-16}\approx 10^{-14}{\text{M}}^{2}. \end{array}$$

And therefore,

(7) $$\begin{array}{c} K_{w}=\left[{\text{H}}^{+}\right]\left[\text{O}{\text{H}}^{-}\right]=10^{-14}. \end{array}$$

The constant ionic product for water ([H^+^][OH^−^]) is equal to 10^−14^ at 25°C.

In pure water, the concentration of hydrogen ions is equal to the concentration of hydroxide ions ([H^+^] = [OH^−^]). At 25°C, both concentrations are equal to 10^−7 ^M. Aqueous solutions are defined as acidic if there is an excess of hydrogen ions over hydroxide ions ([H^+^]>[OH^−^]) or alkaline when there is an excess of hydroxide ions over hydrogen ions ([H^+^]<[OH^−^]), but the ionic product for water ([H^+^][OH^−^]) is always constant in any aqueous solution, regardless of the presence of dissolved solutes. If dissolved substances alter the concentration of either hydrogen ions or hydroxide ions, a concomitant change of the same magnitude must occur in the other ion to maintain constant the ionic product for water. Therefore, the concentration of hydrogen ions rises whenever the concentration of hydroxide ions falls and vice versa, the concentration of hydrogen ions decreases when the level of hydroxide ions increases to maintain the constancy of the anion product for water in aqueous solutions [1–4].

Hence, aqueous solutions such as plasma and urine are electrically neutral and maintain constant the ionic product for water. Variations in the concentration of electrolytes in these solutions drive changes in the state of ionization of water molecules that alter the hydrogen ions concentration in order to preserve electrical neutrality while keeping constant the ionic product for water [1–4].

## 4. Definition of pH

The concentration of hydrogen ions (H^+^) may be expressed in terms of pH, defined as the negative logarithm of the hydrogen ions concentration:

(8) $$\begin{array}{c} \text{pH}=-\log\left[{\text{H}}^{+}\right]=\log\frac{1}{\left[{\text{H}}^{+}\right]}. \end{array}$$

The normal plasma pH is 7.4 (7.35–7.45) [5].

## 5. Definition of p*K*_*a*_

Acids may be defined as substances that increase the concentration of hydrogen ions when added to an aqueous solution, while bases are compounds that decrease the concentration of hydrogen ions (and therefore increase hydroxyl anions concentration) when dissolved in water or aqueous solutions. For instance, hydrochloric acid (HCl) dissociates into H^+^ and chloride (Cl^−^) when dissolved in water, whereas ammonia (NH_3_) becomes protonated when dissolved in water producing ammonium ion (NH_4_^+^).

Each acid has a characteristic tendency to release hydrogen ions in an aqueous solution, according to the reversible reaction:

(9) $$\begin{array}{c} \text{HA}⟺{\text{H}}^{+}+{\text{A}}^{-} \end{array}$$

Any reversible chemical reaction at a specified temperature has a specific equilibrium constant (*K*_*a*_), which defines the final equilibrium concentrations of reactants and products (law of mass action):

(10) $$\begin{array}{c} K_{a}=\frac{\left[{\text{H}}^{+}\right]\left[{\text{A}}^{-}\right]}{\left[\text{HA}\right]}. \end{array}$$

The tendency of an acid to dissociate releasing anions and hydrogen ions may be assessed from this equilibrium constant. The larger the value of the equilibrium constant, the greater the tendency of the acid to dissociate and the stronger the acid.

Bases such as ammonia (NH_3_) attach hydrogen ions when they dissolved in aqueous solutions, to produce protonated molecules such as ammonium ions (NH_4_^+^), according to the reversible reaction:

(11) $$\begin{array}{c} \text{N}{{\text{H}}_{4}}^{+}⟺{\text{H}}^{+}+\text{N}{\text{H}}_{3} \end{array}$$

The equilibrium constant for this reaction is

(12) $$\begin{array}{c} K_{a}=\frac{\left[{\text{H}}^{+}\right]\left[\text{N}{\text{H}}_{3}\right]}{\left[\text{N}{{\text{H}}_{4}}^{+}\right]}. \end{array}$$

The relative strength of a base can be assessed by its equilibrium constant. The lower the equilibrium constant value, the higher the tendency of the base to capture protons and the higher the concentration of the protonated moiety (NH_4_^+^).

The Henderson-Hasselbalch equation is the logarithmic transformation of the equilibrium constant equation:

(13) $$\begin{array}{c} \text{pH}=\text{p}K_{a}+\log\frac{\left[{\text{A}}^{-}\right]}{\left[\text{HA}\right]}\;\text{in}\;\text{the}\;\text{case}\;\text{of}\;\text{an}\;\text{acid}, \end{array}$$

or

(14) $$\begin{array}{c} \text{pH}=\text{p}K_{a}+\log\frac{\left[\text{N}{\text{H}}_{3}\right]}{\left[\text{N}{{\text{H}}_{4}}^{+}\right]}\;\text{in}\;\text{the}\;\text{case}\;\text{of}\;\text{a}\;\text{base}. \end{array}$$

The equilibrium constant may be conveniently stated in the form of p*K*_*a*_, which is defined as the negative logarithm of *K*_*a*_:

(15) $$\begin{array}{c} \text{p}K_{a}=-\log K_{a}=\log\frac{1}{K_{a}}. \end{array}$$

The p*K*_*a*_ reflects the relative strength of an acid. The stronger the acid, the greater its tendency to dissociate releasing anions and hydrogen ions and the lower its p*K*_*a*_ value. The p*K*_*a*_ of a base gauges its tendency to join hydrogen ions. The stronger the base, the greater its tendency to bind protons and the higher its p*K*_*a*_ value.

When the concentration of the undissociated moiety (HA, NH_4_^+^) is equal to the concentration of the dissociated moiety (A^−^, NH_3_), pH equals p*K*_*a*_, as log⁡⁡1 = 0. Therefore, the p*K*_*a*_ of an acid or a base is the pH at which the concentration of dissociated and undissociated forms is the same.

## 6. Strong Acids and Bases

When the p*K*_*a*_ of an acid is lower than the pH of the aqueous solution in which it is dissolved, the acid dissociates releasing hydrogen ions, being transformed into an anion. Strong acids have low p*K*_*a*_ values and completely dissociate in aqueous solutions, yielding strong anions. Acids with p*K*_*a*_ value of approximately 4 or less generate strong anions at physiologic pH values. Many organic acids existing in the human body possess p*K*_*a*_ values around 4 and therefore appear predominantly in their anionic form, such as bicarbonate, lactate, pyruvate, citrate, acetoacetate, and *β*-hydroxybutyrate (Table 1).

When the pH of the aqueous solution is lower than the p*K*_*a*_ value of a dissolved base, the base captures hydrogen ions and becomes protonated. Strong bases have high p*K*_*a*_ values and therefore they remain protonated at physiologic plasma pH. For instance, the p*K*_*a*_ value for the pair NH_3_/NH_4_^+^ is 9.3, indicating that virtually only the protonated moiety (NH_4_^+^) is present in the human body.

Weak acids and bases are those with p*K*_*a*_ values closer to approximately 7. At physiological pH values, their degree of dissociation or protonation, respectively, is variable. Weak anions in the human body include the pair dihydrogen phosphate/monohydrogen phosphate (H_2_PO_4_^−^/HPO_4_^2−^) and urate (Table 1).

## 7. Physiological Relationship between Carbon Dioxide and Plasma Bicarbonate

Plasma bicarbonate is a circulating anion that contributes substantially to the plasma negative charge in quantitative terms and therefore plays a role in defining plasma pH. Unlike other plasma ions, bicarbonate concentration is determined by the lung ventilatory activity besides the kidney handling of bicarbonate. In addition, several isoenzymes of carbonic anhydrase are ubiquitously expressed in the human body and catalyze the reversible conversion of carbon dioxide into bicarbonate [6].

In humans, carbon dioxide (CO_2_) is an end product of cell metabolism continuously incorporated into the tissue capillaries and normally removed from the body by the expired air [7].

**Figure 1:**
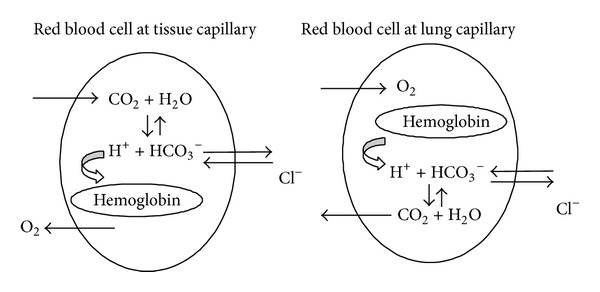
Carbon dioxide transport in blood.

Carbon dioxide formed during cell metabolism diffuses across the plasma membrane into the tissue capillary network, being transported by blood in a number of ways. Approximately 5% of the carbon dioxide remains as a gas in the aqueous phase of blood, being measured as the partial pressure of carbon dioxide (pCO_2_). An even smaller proportion of carbon dioxide binds to plasma proteins. Most of the carbon dioxide (90–95%) arriving in capillary blood diffuses into the red blood cells, where it is hydrated to bicarbonate (HCO_3_^−^) by the cytosolic enzyme carbonic anhydrase II, generating hydrogen ions that bind to oxyhemoglobin. As a result, oxygen (O_2_) is released from oxyhemoglobin and leaves the erythrocyte, reaching the tissue cells. Bicarbonate formed inside the red blood cells by carbonic anhydrase II leaves these cells towards the plasma in exchange for chloride through the plasma membrane transporter anion exchanger-1 (AE1). Erythrocytes with protonated deoxyhemoglobin formed in the tissue capillaries travel to the lungs, where the uptake of oxygen transforms deoxyhemoglobin into oxyhemoglobin, releasing hydrogen ions that are combined with bicarbonate diffusing back from plasma by carbonic anhydrase II, generating water and carbon dioxide, which is exhaled as a gas (Figure 1) [8, 9].

Carbonic anhydrase is a zinc-containing enzyme that catalyzes the reversible hydration of carbon dioxide (CO_2_) to bicarbonate (HCO_3_^−^) and a proton (H^+^). The molecule of carbonic anhydrase contains a conic cavity at the bottom of which is the zinc atom. The reaction catalyzed by the enzyme involves two steps. First, carbon dioxide reacts with hydroxide ions (OH^−^) from water leading to the formation of HCO_3_^−^, which is displaced by a water molecule. The second step involves the transfer of an H^+^ from the water molecule to one histidine residue, reforming OH^−^ [6]. The net result of the carbonic anhydrase II reaction is

(16) $$\begin{array}{c} {\text{CO}}_{2}+{\text{H}}_{2}\text{O}⟷{{\text{HCO}}_{3}}^{-}+{\text{H}}^{+} \end{array}$$

In human erythrocytes, plasma membrane AE1 and cytosolic carbonic anhydrase II form a functional complex [9, 10].

Blood carbon dioxide content is predominantly determined by pulmonary ventilatory activity.

An increment in the respiratory rate (hyperventilation) results in carbon dioxide elimination leading to hypocapnia, whereas a reduction in the respiratory rate (hypoventilation) promotes carbon dioxide retention (hypercapnia) [7]. Since plasma bicarbonate is the principal way of transportation of carbon dioxide in blood, plasma bicarbonate level increases as carbon dioxide rises, while plasma bicarbonate concentration falls as carbon dioxide declines. Both carbon dioxide and plasma bicarbonate reflect the ventilatory status. Hypoventilation results in hypercapnia and a rise in plasma bicarbonate concentration, while hyperventilation leads to hypocapnia and a subsequent reduction in the plasma bicarbonate level [11, 12].

A quantitative relationship between carbon dioxide and plasma bicarbonate has been demonstrated in a number of situations, further highlighting their physiological relationship.

In patients with chronic hypercapnic respiratory failure, the plasma bicarbonate level increases by 5.1 mM for each 10 mmHg increase in the arterial pCO_2_ [13]. In panic disorder patients with hyperventilation attacks, for each decrease of 1 mmHg in arterial pCO_2_, plasma bicarbonate level decreases by 0.41 mEq/L [14]. The same quantitative relationship between arterial pCO_2_ and plasma bicarbonate concentration has been found in healthy volunteers undergoing simulated altitude: for each decrease of 1 mmHg in the arterial pCO_2_, the plasma bicarbonate concentration decreases by 0.41 mM [7]. A close linear relationship between arterial pCO2 and plasma bicarbonate also exists in patients with metabolic alkalosis due to diuretic use or vomiting, in whom the arterial pCO_2_ increases 1.2 mmHg for each 1.0 mM increment in plasma bicarbonate level [15]. In patients with metabolic acidosis, compensatory hyperventilation reduces plasma carbon dioxide and consequently plasma bicarbonate. In most of these patients, the decrease in plasma bicarbonate concentration may be predicted from the arterial pCO_2_ by the Winter's equation [16, 17].

A close linear relationship between arterial pCO2 and plasma bicarbonate also exists in patients with metabolic alkalosis due to diuretic use or vomiting, in whom the arterial pCO2 increases 1.2 mmHg

(17) $$\begin{array}{c} \text{arterial}\;{\text{pCO}}_{2}=1.5\times \left[{{\text{HCO}}_{3}}^{-}\right]+8. \end{array}$$

Furthermore, a correlation between end-tidal pCO_2_ and plasma bicarbonate concentration has been demonstrated in a number of studies. Capnometry is the measurement of the pCO_2_ at the end of an exhaled breath, which is termed end-tidal carbon dioxide. In nonintubated patients presenting to an emergency department [18] and in patients with diabetic ketoacidosis [19], end-tidal carbon dioxide correlates with arterial pCO_2_. Accordingly, end-tidal pCO_2_ monitoring may be used as surrogate for arterial pCO_2_ and prevent from cumbersome arterial blood gases extraction [18]. In addition, end-tidal pCO_2_ is correlated with serum bicarbonate level and capnography has been used as noninvasive accurate estimate of plasma bicarbonate concentration in patients with diabetic ketoacidosis [17, 20], among children with gastroenteritis [21], and in patients presenting to the emergency department [22]. In children with gastroenteritis, end-tidal pCO_2_ values greater than 34 mmHg ruled out plasma bicarbonate concentration lower than 15 mmol/L [21].

The relationship between carbon dioxide and bicarbonate is additionally underlined by the fact that the infusion of sodium bicarbonate increases carbon dioxide production and arterial pCO_2_, as it was first documented in 1956 [23, 24].

In addition to pulmonary function, plasma bicarbonate concentration is determined by the kidney tubule, which may recover filtered bicarbonate or secrete bicarbonate into the urine depending on physiological requirements. Most bicarbonate filtered at the glomerulus is normally reclaimed by the proximal convoluted tubule through mechanisms that are being elucidated and involve both basolateral and apical ion transporters and isoenzymes of carbonic anhydrase. In addition to its ability to bicarbonate reabsorption by the proximal tubule, normal human kidney has a large capacity to excreting bicarbonate in more distal segments of the nephron. The collecting ducts possess at least two bicarbonate transporters in humans, the kidney isoform of the AE1 at the basolateral membrane and pendrin on the apical membrane. The enzyme carbonic anhydrase is also necessary to bicarbonate handling in this portion of the nephron.

In the proximal tubule, the sodium-bicarbonate (Na^+^-HCO_3_^−^) cotransporter NBCe1 mediates basolateral electrogenic sodium-coupled bicarbonate transport and is required for bicarbonate (and sodium) recovery on this segment of the nephron [25]. Additionally, sodium/proton exchangers (NHEs) that mediate exit of hydrogen ions from cells with uptake of sodium [26], vacuolar H^+^-ATPase that mediates the ATP-dependent transport of protons [27], and Na^+^-K^+^ ATPase [28] are involved in bicarbonate reabsorption in the proximal tubule. Activity of the enzyme carbonic anhydrase II is also required to bicarbonate reabsorption in this portion of the nephron [29].

In the ascending segment of the loop of Henle, the sodium-potassium-chloride (Na^+^-K^+^-2Cl^−^) cotransporter located at the apical membrane drives the electroneutral reabsorption of sodium, potassium, and chloride. This cotransporter is furosemide-sensitive, being inhibited by the administration of this diuretic. Blockade of the Na^+^-K^+^-2Cl^−^ cotransporter enhances sodium delivery to the collecting duct, where sodium is reabsorbed without chloride by the epithelial sodium channel (ENaC) [30, 31]. In addition, dysfunction of the Na^+^-K^+^-2Cl^−^ cotransporter induces plasma volume contraction and subsequent activation of the renin-angiotensin-aldosterone (RAA) system that amplifies sodium reabsorption without chloride through the ENaC [30, 32, 33].

In the early distal convoluted tubule, the sodium-chloride (Na^+^-Cl^−^) cotransporter is located at the apical membrane and links sodium and chloride reabsorption. This cotransporter is thiazide-sensitive, being inhibited by the administration of hydrochlorothiazide. Blockade of the Na^+^-Cl^−^ cotransporter increases sodium delivery to the collecting duct where uptake of sodium takes place without chloride by the ENaC [30, 32].

In the collecting duct, the ENaC resides on the apical membrane of principal cells and mediates sodium reabsorption without chloride, increasing the lumen electronegativity and creating an electrical gradient that promotes potassium and hydrogen ions secretion into the urine. The H^+^-ATPases that secrete protons into the tubular lumen are functionally coupled to the basolateral kidney isoform of the AE1 that exchanges bicarbonate and chloride. The cytosolic enzyme carbonic anhydrase II provides the substrate for both transporters by catalyzing the hydration of carbon dioxide to bicarbonate and hydrogen ions. Bicarbonate is reabsorbed into the blood in exchange for chloride that is excreted into the urine, while hydrogen ions are secreted into the tubular lumen by the H^+^-ATPase. Therefore, sodium reabsorption by the ENaC is linked to bicarbonate reclamation in exchange for chloride [34–36].

The ENaC is amiloride-sensitive and administration of this diuretic blocks the channel. As a consequence, sodium reabsorption with concomitant chloride excretion is suppressed and therefore sodium is lost in excess of chloride in the urine resulting in plasma retention of chloride relative to sodium [34–36].

The ENaC is primarily regulated by flow rate and aldosterone. Blockade of sodium reabsorption earlier in the nephron, either at the Na^+^-K^+^-2Cl^−^ cotransporter on the loop of Henle or the Na^+^-Cl^−^ cotransporter on the distal tubule, enhances urinary flow and sodium delivery to the collecting duct where uptake of sodium takes place via the ENaC, while bicarbonate is reabsorbed in exchange for chloride. In addition, mineralocorticoids such as aldosterone stimulate sodium reabsorption in the collecting duct through activation of the ENaC and therefore allow bicarbonate recovery and chloride loss in urine. Aldosterone antagonists spironolactone and eplerenone inhibit aldosterone action and therefore suppress ENaC activity, mimicking the effects of amiloride. Sodium is wasted in urine in excess of chloride, while the urinary excretion of potassium and hydrogen ions is inhibited [34–36].

Pendrin is a chloride/bicarbonate (Cl^−^/HCO_3_^−^) exchanger positioned at the apical membrane of intercalated cells of the cortical collecting ducts that accounts for the secretion of bicarbonate into the tubule lumen in exchange for chloride reabsorption, modulating bicarbonate handling by the kidney [37–40].

## 8. The Acid-Base Balance in Humans

In human beings, normal metabolism in tissue cells continuously produces carbon dioxide, cations such as ammonium, and anions such as lactate (L-lactate and D-lactate), ketone bodies (acetoacetate and *β*-hydroxybutyrate), phosphate (H_2_PO_4_^−^/HPO_4_^2−^), and sulphate (SO_4_^2−^). In addition, other charged molecules circulate in human plasma, including negative charges such as albumin and positive charges such as immunoglobulins. Anomalous plasma ions may be derived from exogenous substances, including cations such as lithium and anions such as formate generated from methanol. All of them may potentially change the plasma pH when modifications in their concentration alter the normal relative proportion of plasma anions and cations. According to the electroneutrality principle and the constancy of the ionic product for water, the plasma concentration of hydrogen ions depends on the plasma level of other ions and variations in the plasma ionic composition predictably lead to a change in plasma pH. Plasma acidosis (plasma pH less than 7.35) is caused by an excess of plasma anions relative to cations, which may be due to either an elevation of plasma anions out of proportion of cations (such as hyperchloremia with no variation in plasma sodium) or a fall in plasma cations (such as hyponatremia with no change in plasma chloride). The relative excess of anions over cations creates an electrical imbalance that requires the implementation of adaptive mechanisms. To regain electrical neutrality, the remaining negative charge must be reduced and this is achieved by a fall in the hydroxide anions concentration. In turn, the drop of hydroxide anions causes an increase in hydrogen ions of the same magnitude to maintain constant the ionic product for water, decreasing plasma pH. By contrast, plasma alkalosis (plasma pH greater than 7.45) is brought about by a relative reduction of plasma anions relative to cations, which may be due to either a decrease in plasma anions out of proportion of cations (such as hypochloremia with no change in plasma sodium) or an increase in plasma cations (hypernatremia with no variation in plasma chloride). To comply with the electroneutrality principle, the fall of plasma anions out of proportion of cations is compensated by raising hydroxide anions. The increment of hydroxide ions implies a fall of the same magnitude in plasma hydrogen ions to maintain constant the ionic product for water, increasing plasma pH. Therefore, modifications in the plasma ionic composition that jeopardize electrical balance drive adjustments in water dissociation in order to recover electrical neutrality and maintain the constancy of the ionic product for water, which ultimately alter the plasma concentration of hydrogen ions [1–4].

Quantitatively, the main anions normally circulating in human plasma are chloride and bicarbonate, while the most abundant plasma cation is sodium. Alterations in the concentration of these ions that modify their relative proportion inducing an electrical imbalance are the predominant cause of acid-base disorders. Pulmonary function modifies carbon dioxide elimination and consequently plasma bicarbonate concentration, as this anion is the major way of carrying carbon dioxide in blood. Therefore, respiratory acid-base disorders are associated with primary changes in plasma bicarbonate concentration, while metabolic acid-base disorders are caused by primary alterations in other plasma ions, mainly chloride and sodium. In addition, modifications in the pulmonary ventilatory rate are critical adaptive mechanisms implemented to mitigate fluctuations in blood pH when plasma anions accumulate (acidosis) or are deficient (alkalosis) relative to cations. Metabolic acidosis induces hyperventilation in order to decrease the plasma concentration of bicarbonate, whereas metabolic alkalosis induces hypoventilation to raise plasma bicarbonate concentration. Both compensatory mechanisms may be life-saving when severe deviations from normal pH take place [40].

### 8.1. Metabolic Acidosis

Metabolic acidosis is caused by an excess of plasma anions relative to cations that may be generated either by plasma accumulation of anions or by marked reduction of plasma cations (sodium ion) which similarly leads to relative excess of negative ions. The electrical imbalance driven by the accumulation of anions out of proportion of cations induces a fall in the hydroxide anions concentration, which implies an increase in the hydrogen ions concentration, reducing the plasma pH. In addition, a compensatory increase in the ventilatory rate achieves a reduction of the plasma bicarbonate concentration. The decline in plasma bicarbonate contributes to restricting the fall in hydroxide anions, limiting the increase in hydrogen ions.

The anions most frequently encountered in conditions leading to metabolic acidosis are chloride (hyperchloremic acidosis), L-lactate (L-lactic acidosis), and the ketone bodies acetoacetate and *β*-hydroxybutyrate (ketoacidosis). D-lactate and other organic anions such as formate may also be a cause of metabolic acidosis when their concentration is elevated. The accumulation of anions other than chloride resulting in plasma acidosis is called anion gap acidosis. Sometimes the same causative factor may produce acidosis by different mechanisms. For instance, toluene abuse has been associated with either hyperchloremic or anion gap acidosis [41, 42]. Distal renal tubular acidosis is the predominant cause of hyperchloremic acidosis secondary to toluene poisoning [43], while lactic acidosis [44] and ketoacidosis [45] have been found in cases of toluene intoxication with anion gap acidosis.

#### 8.1.1. Hyperchloremic (Nonanion Gap) Acidosis

An increase in the plasma concentration of chloride out of proportion of sodium causes hyperchloremic acidosis. The relative increase in plasma chloride may be due to either exogenous administration of chloride in excess of sodium or a decrease in the plasma sodium concentration with no change in the chloride level. In addition, either intestinal or renal loss of fluid with low chloride concentration relative to sodium induces hyperchloremic acidosis, owing to plasma retention of chloride in excess of sodium [46, 47]. The factor that effectively modulates plasma pH is the relative proportion between sodium and chloride concentrations. When an elevation in plasma chloride occurs simultaneously with an increase in plasma sodium, hyperchloremia is not associated with acidosis, as there is no electrical imbalance to compensate. By contrast, a patient with normal plasma chloride concentration and hyponatremia develops acidosis due to relative hyperchloremia, as there is an increment in plasma chloride relative to sodium [2–4].

Causes of hyperchloremic acidosis include marked hyponatremia with normochloremia, ureteral diversion into the intestinal tract, the administration of exogenous substances that provide excess chloride relative to sodium, and loss of relatively chloride-free fluid, either intestinal (diarrhea) or urinary (renal tubular acidosis).

Metabolic acidosis has been documented associated with a decrease in the plasma sodium concentration out of proportion of plasma chloride [48, 49].

Ureteral diversion into the intestinal tract (ureterosigmoidostomy) causes hyperchloremic acidosis due to intestinal reabsorption of urinary chloride that leads to relative hyperchloremia [47].

Plasma acidosis occurs after normal saline (0.9% sodium chloride solution) infusion into the blood stream during fluid resuscitation attempts [50]. The concentration of sodium ion and chloride ion in normal saline is the same (154 mM), while plasma sodium concentration is approximately 40 mM greater than plasma chloride level. Therefore, the infusion of normal saline into the blood stream increases proportionally more the concentration of chloride than that of sodium, resulting in an excess of plasma chloride relative to sodium. Similarly, hyperchloremic acidosis is produced in preterm infants fed with milk or parenteral nutrition providing excess chloride relative to sodium [51].

Diarrhea and pancreatic or biliary fistulas induces hyperchloremic acidosis due to the waste of intestinal fluid rich in sodium and relatively chloride-free, as both pancreatic and biliary secretions are rich in sodium bicarbonate [47, 52, 53].

Likewise, renal tubular acidosis (RTA) causes hyperchloremic acidosis owing to the loss of urine containing low chloride concentration relative to sodium. Either proximal or distal dysfunction of the kidney tubule may be associated with urinary loss of sodium bicarbonate in excess of chloride.

Proximal RTA is caused by congenital or acquired disorders of the proximal convoluted tubule associated with defective sodium and bicarbonate reabsorption that cause urinary loss of sodium out of proportion of chloride [54]. Among congenital disorders, mutations in* SLC4A4*, the gene encoding the sodium-bicarbonate cotransporter NBCe1, cause proximal RTA [25]. Autoimmune diseases that damage the proximal convoluted tubule may produce acquired proximal RTA [55]. Patients with proximal RTA are able to reclaim filtered bicarbonate when the filtered load is low and therefore their urine may be acid in presence of plasma acidosis, as distal acidification mechanisms remain intact [46]. Proximal RTA may be isolated or a part of a more generalized tubule defect, named Fanconi syndrome, characterized by elevated urinary excretion of solutes like phosphate, uric acid, potassium, glucose, amino acids, and low-molecular-weight proteins [55].

Distal RTA is a heterogeneous group of inherited and acquired disorders characterized by the inability to acidify the urine in the collecting duct, where sodium reabsorption through the amiloride-sensitive ENaC is linked to bicarbonate recovery and chloride excretion in urine. Unlike proximal RTA, patients with distal RTA maintain inappropriately alkaline urine in the presence of hyperchloremic acidosis [46, 56]. Tubulointerstitial nephropathies that injure collecting ducts, hypoaldosteronism states, and the use of amiloride or aldosterone antagonists such as spironolactone and eplerenone induce urinary loss of sodium in excess of chloride that results in plasma chloride retention relative to sodium and hyperchloremic acidosis [35].

Congenital or acquired deficiency of carbonic anhydrase II leads to RTA often combined proximal and distal, as the enzyme is present in both segments of the kidney tubule. Besides RTA, congenital deficiency of carbonic anhydrase II is characterized by cerebral calcification, osteopetrosis, mental retardation, and growth failure [29, 57]. The administration of carbonic anhydrase inhibitors such as acetazolamide induces hyperchloremic acidosis due to urinary excretion of sodium without chloride [58].

#### 8.1.2. Metabolic Acidosis Associated with Unmeasured Anions (Anion Gap Metabolic Acidosis)

Anion gap metabolic acidosis is caused by accumulation of plasma anions other than chloride. These anions are not usually measured in the electrolyte panel, being called unmeasured anions. The most frequent unmeasured anions that cause metabolic acidosis are L-lactate (L-lactic acidosis), D-lactate (D-lactic acidosis), *β*-hydroxybutyrate and acetoacetate (ketoacidosis), organic acids such as pyroglutamate from acetaminophen, anions derived from metabolism of some alcohols, phosphate, and sulphate derived from dietary animal protein.

L-lactic acidosis may be secondary to congenital or acquired mitochondrial respiratory chain dysfunction, including tissue hypoxia [59], carbon monoxide poisoning [60], cyanide poisoning [61] and drugs such as linezolid, phenformin, and stavudine [62–64]. In addition, pyruvate dehydrogenase deficiency [65], defective gluconeogenesis pathway [66], thiamine (vitamin B1) deficiency [67], malignancy [68], liver disease [69], sepsis [70], asthma [71], fructose infusion [72], and ingestion of ethanol [73] may produce L-lactic acidosis.

D-lactic acidosis occurs following small bowel resection (short bowel syndrome), intestinal bypass surgery for obesity [74], and administration of high doses of propylene glycol [75]. In addition, patients with diabetic ketoacidosis show elevated plasma D-lactate level compared to diabetic patients without ketoacidosis [76].

A variety of conditions may increase the production of ketone bodies (*β*-hydroxybutyrate and acetoacetate), including a ketogenic diet [77], starvation or prolonged fasting [78], uncontrolled type 1 and type 2 diabetes mellitus (diabetic ketoacidosis) [79], and ethanol metabolism (alcoholic ketoacidosis) [80].

Pyroglutamic acid or 5-oxoproline is an intermediary in the *γ*-glutamyl cycle, which facilitates the transport across cellular membranes of amino acids that participate in the synthesis of glutathione. Pyroglutamic acidosis is an anion gap metabolic acidosis that may be either congenital, due to an autosomal recessive deficiency of glutathione synthetase (5-oxoprolinase) or acquired, due to ingestion of paracetamol and more rarely other medications, including vigabatrin, netilmicin, and flucoxacillin [81]. Pyroglutamic acidosis is characterized by elevated urinary excretion of 5-oxoproline [82].

Intoxication by some alcohols, including ethanol [73, 80], methanol, [83], and propylene glycol [75, 84] induces anion gap acidosis associated with L-lactic acidosis and accumulation of anions derived from alcohol metabolism, including ketone bodies (ethanol), formate (methanol), D-lactate (propylene glycol), and oxalate (ethylene glycol) [85, 86].

The diagnosis of anion gap acidosis is not straightforward, as the detection of anions not usually measured by the plasma chemical profile (unmeasured anions) has to be indirectly performed via some equations, such as the anion gap, the chloride/sodium ratio, and the strong ion gap [1, 87].

*Serum Anion Gap.* According to the electroneutrality principle, the sum of cations must be equal to the sum of anions in human plasma:

(18) [Na+]+[K+]+[unmeasured  cations]  =[Cl−]+[HCO3−]+[unmeasured  anions].

Therefore,

(19) [Na+]+[K+]−([Cl−]+[HCO3−])=[unmeasured  anions]−[unmeasured  cations].

The serum anion gap can be calculated with the following equation:

(20) Serum  anion  gap⁡ (mEq/L)=([Na+]+[K+])−([Cl−]+[HCO3−]).

The most commonly used formula for the calculation of the serum anion gap excludes potassium concentration:

(21) Serum  anion  gap⁡ (mEq/L)=[Na+]−([Cl−]+[HCO3−]),

or

(22) Serum  aniongap (mEq/L)=[unmeasured  anions]−[unmeasured  cations].

Normally, unmeasured anions outnumber unmeasured cations in human plasma and the anion gap is positive. The normal value for the calculated serum anion gap is 8–16 mEq/L.

The anion gap provides an estimation of the unmeasured anions in the plasma, including albumin and phosphate. As serum albumin is an unmeasured anion, a decrease in plasma albumin concentration lowers the anion gap. It has been estimated that each g/L decrease in serum albumin diminishes the anion gap by 0.25 mEq/L (for each 1 g/dL descent in serum albumin, the anion gap would decrease by 2.5 mEq/L). Failure to correct the calculated anion gap for the plasma albumin concentration may underestimate the true anion gap. A normal unadjusted anion gap does not exclude the presence of unmeasured anions in patients with hypoalbuminemia [88, 89]. Therefore, correction of the calculated anion gap for serum albumin improves the usefulness and accuracy of this parameter. The following equation may be used, expressing albumin level in g/L: adjusted serum anion gap = observed anion gap + 0.25 × ([normal alb]−[observed alb]). The factor is 2.5 if albumin concentration is given in g/dL [87, 89].

An increase in the albumin-adjusted anion gap denotes the presence of unmeasured anions, such as phosphate, sulphate, lactate, ketone bodies, formate, and oxalate, provided that the level of cations is not altered. A low anion gap occurs when there is a significant increase in unmeasured cations, as in lithium intoxication and multiple myeloma (accumulation of cationic paraproteins). Bromide is identified as chloride in some analyzers producing spurious hyperchloremia and a marked reduction in the anion gap [47, 90].

*Chloride/Sodium Ratio.* In response to metabolic acidosis due to accumulation of unmeasured anions, plasma chloride level decreases relative to sodium, reducing the chloride/sodium ratio ([Cl^−^]/[Na^+^]), although the absolute value of plasma chloride may remain normal. The chloride/sodium ratio may be used as surrogate to detect unmeasured anions in patients with metabolic acidosis. A [Cl^−^]/[Na^+^] ratio lower than 0.75 identifies the presence of unmeasured anions with a likelihood ratio similar to the anion gap. Conversely, a high ratio (>0.79) excludes plasma unmeasured anions in patients with metabolic acidosis [91]. In critically ill neonates, the chloride/sodium ratio has been efficiently used as a tool to evaluate raised unmeasured anions. In these patients, a negative correlation between the chloride/sodium ratio and corrected anion gap is observed [92]. 

*Apparent Strong Ion Difference.* In normal human plasma, the strong cations usually measured in chemical profiles outnumber the usually measured strong anions and the difference between them has been named apparent strong ion difference (SIDa) [1]. Consider

(23) SIDa (mEq/L)=[usually  measured  strong  cations]  −[usually  measured  strong  anions],SIDa (mEq/L)=([Na+]+[K+]+[Ca2+]+[Mg2+])  −([Cl−]+[lactate−]).

In normal plasma, the SIDa value is 38 to 42 mEq/L. The increase of usually measured strong anions out of proportion of usually measured strong cations reduces the apparent strong ion difference, being a major cause of acidosis, termed SIDa acidosis. SIDa acidosis may be due to hyperchloremic acidosis or lactic acidosis. If lactate concentration is normal, SIDa acidosis is equivalent to hyperchloremic acidosis [1, 2, 4]. 

*Plasma Sodium Concentration Minus Plasma Chloride Concentration Difference (Sodium-Chloride Effect).* As sodium and chloride are the ions with the highest plasma concentration, the difference between them ([Na^+^]−[Cl^−^]) can be used as surrogate for the apparent SID [2]. In critically ill patients, there is a positive correlation between the [Na^+^]−[Cl^−^] difference and the SIDa and the [Na^+^]−[Cl^−^] difference reveals SIDa acidosis with high accuracy [3, 92]. As the [Na^+^]−[Cl^−^] difference predicts the SIDa, the SIDa calculation may be substituted for the [Na^+^]−[Cl^−^] effect in the diagnosis of acid-base disorders [4]. 

*Effective Strong Ion Difference.* The apparent SID has to be counterbalanced by quantitatively equal negative charges to preserve plasma electroneutrality. The negative charges that offset the apparent SID are mostly contributed by albumin, phosphate, and bicarbonate, and the sum of all of them has been called the effective strong ion difference (SIDe) [1, 87]:

(24) $$\begin{array}{c} \text{SIDe}\;\left(\text{mEq}/\text{L}\right)=\left[{{\text{HCO}}_{3}}^{-}\right]+\left[{\text{Alb}}^{-}\right]+\left[{\text{Pi}}^{-}\right]. \end{array}$$

*Strong Ion Gap.* The difference between the apparent and the effective strong ion differences is termed the strong ion gap (SIG):

(25) $$\begin{array}{c} \text{SIG}\;\left(\text{mEq}/\text{L}\right)=\;\text{SIDa}-\text{SIDe}. \end{array}$$

In normal plasma with no excess unmeasured anions, apparent and effective strong ion differences are equal and therefore the SIG is zero. The SIG becomes positive when there are unidentified anions in plasma, as the presence of unmeasured anions in plasma usually induces a decrease in the effective SID (due to a fall in plasma bicarbonate concentration) and an increase in the apparent SID (due to a decrease in plasma chloride level), making positive the difference between SIDa and SIDe. Therefore, similarly to the anion gap, a positive SIG detects the presence of unmeasured anions [1]. It has been shown that the anion gap corrected for albumin and lactate can be used as surrogate for the SIG, avoiding its cumbersome calculation. An adjusted anion gap greater than 8 mEq/L accurately predicts SIG acidosis [4].

### 8.2. Respiratory Acidosis

Neurological and respiratory disorders, such as chronic obstructive pulmonary disease (COPD), may impair carbon dioxide elimination leading to carbon dioxide retention and consequently to an increase in the plasma bicarbonate level, as bicarbonate is the predominant way in which carbon dioxide is transported in plasma [93].

In patients with chronic respiratory failure, carbon dioxide retention and raised plasma bicarbonate level are not usually associated with substantial deviations of the plasma pH and near-normal blood pH values have been repeatedly observed, likely owing to the fact that the urinary chloride excretion rises with increasing carbon dioxide retention, leading to a fall in the plasma chloride concentration with no variation in plasma sodium [11, 93]. In stable outpatients with chronic hypercapnic respiratory failure from COPD, the arterial pCO_2_ ranged from 45 to 77 mmHg, whereas the pH ranged from 7.37 to 7.44. The overall average pH was 7.40, while the overall mean pCO_2_ was 58 mmHg. The lowest observed pH was 7.37 and a pH lower than 7.38 was rarely encountered. Despite having arterial pCO_2_ levels as high as 77 mmHg, all of the patients maintained their pH at 7.37 or greater and more than 80% had a pH of 7.38 or greater. In addition, an increase of 10 mmHg in the pCO_2_ was associated with an increase of 5.1 mM in the plasma bicarbonate level and a decrease of only 0.014 in the plasma pH. Therefore, in a stable patient with a pCO_2_ of 55 mmHg, the predicted plasma pH would be 7.40 with a bicarbonate level of 33 mM [13]. In patients with COPD and chronic hypercapnia, the plasma bicarbonate concentration is elevated and the blood pH is only slightly reduced at 7.37. In these patients, a significant decrease in plasma chloride concentration is observed with no modification in plasma sodium [11]. Among hospitalized hypercapnic patients, a normal arterial pH was seen in 87% of the patients with pCO_2_ between 46 and 55 mmHg [12]. The arterial pH on admission to the hospital in patients with chronic respiratory failure was not significantly away from normal with a range from 7.37 to 7.41 [94].

In stable patients with cystic fibrosis and severe pulmonary involvement, the fall in plasma chloride concentration is more pronounced than in patients with COPD with comparable airway obstruction. Accordingly, the frequency of metabolic alkalosis in patients with cystic fibrosis is significantly greater than the frequency of this acid-base disorder in patients with COPD. The pathogenic mechanism that induces hypochloremia and metabolic alkalosis in patients with cystic fibrosis has not been elucidated, but defective cystic fibrosis transmembrane conductance regulator function with abnormal electrolyte transport within the gastrointestinal tract or the kidney likely plays a role [95].

Patients with acute respiratory failure also show hypercapnia, elevated plasma bicarbonate concentration, and reduced plasma chloride concentration with no variation in plasma sodium. Unlike patients with chronic pulmonary disorders, patients with acute respiratory failure tend to display more acidic plasma pH in some studies [96], but plasma acidosis in these patients is likely related to metabolic causes of acidosis, such as L-lactic acidosis due to *β*_2_-adrenergic agonists and the increasing rate and depth of breathing in patients with acute respiratory failure may represent compensatory hyperventilation to metabolic acidosis rather than worsening airway obstruction [97, 98].

In patients with acute respiratory failure of neuromuscular cause, a wide range of plasma pH has been observed, from 7.13 to 7.51, with a mean value of 7.35 and a median value of 7.38. The average plasma bicarbonate concentration in these patients is 29.8 mM (19–49) and the average pCO_2_ is 55 mmHg (26–100) [99].

### 8.3. Metabolic Alkalosis

Metabolic alkalosis is caused by a reduction in the plasma chloride level out of proportion of plasma sodium that may occur either by loss of chloride exceeding sodium or by a marked increase in plasma sodium with normochloremia which likewise results in a relative deficiency of chloride. The fall in the plasma chloride concentration relative to plasma sodium causes metabolic alkalosis by driving adjustments in the state of dissociation of water to preserve both plasma electroneutrality and the constancy of the ionic product for water. To maintain electrical neutrality, the decline in plasma chloride induces an increase in hydroxide anions that in turn implies a drop in the hydrogen ions concentration of the same magnitude to ensure the constancy of the ionic product for water, increasing the pH. In addition, a compensatory decrease in the ventilatory rate (hypoventilation) is a critical adaptive mechanism that raises the plasma bicarbonate concentration, preventing the excessive increment of hydroxide anions that generate alkalosis [15, 40].

The predominant cause of metabolic alkalosis is either gastrointestinal or urinary loss of chloride that induces chloride depletion out of proportion of sodium, but a similar relative descent in plasma chloride may be generated by an increment in plasma sodium without modification of plasma anions. Indeed, metabolic alkalosis regularly occurs in critically ill patients developing hypernatremia without concomitant hyperchloremia [100, 101].

Causes of metabolic alkalosis due to gastrointestinal loss of chloride exceeding sodium include vomiting, gastric suction, and loss of intestinal fluid rich in chloride in rare cases of congenital chloridorrhea, a chloride-rich diarrhea caused by loss of function mutations in the* downregulated in adenoma* gene leading to a defect in the apical membrane chloride/bicarbonate exchanger in the distal ileum and colon [31, 40, 52, 102].

Metabolic alkalosis associated with chloride waste in the urine out of proportion of sodium is usually secondary to excess reabsorption of sodium by the ENaC (with attendant excessive chloride excretion), which may occur in a number of conditions, including furosemide use, Bartter syndrome, administration of hydrochlorothiazide, Gitelman syndrome, excess mineralocorticoid activity (primary or secondary aldosteronism), and high dose glucocorticoids. Conditions that lead to apparent mineralcortidoid excess, such as Liddle syndrome and deficiency of 11*β*-hydroxysteroid dehydrogenase, also exhibit metabolic alkalosis. In addition, this acid-base disorder may be a feature of Pendred syndrome.

Urinary chloride level is low (<10 mEq/L) in patients with gastrointestinal loss of chloride or prior diuretic use, while a high concentration of chloride in the urine (>10 mEq/L) suggests loss of chloride by the kidney including continued diuretic use [30, 102].

Blockade of the apical membrane Na^+^-K^+^-2Cl^−^ cotransporter located at the ascending limb of the Henle's loop increases the amount of sodium that reaches the collecting duct, where sodium is reclaimed without chloride by the ENaC, resulting in metabolic alkalosis. Secondary hyperaldosteronism associated with activation of the RAA due to plasma volume contraction intensifies sodium reabsorption by the ENaC with chloride waste in the urine [30–33]. Furosemide administration induces acquired inhibition of this cotransporter, whereas congenital blockade is due to mutations in the gene encoding the transporter (Bartter syndrome) [30, 31].

Similarly, blockade of the apical Na^+^-Cl^−^ cotransporter in the distal tubule causes metabolic alkalosis by enhancing sodium release to the collecting duct and inducing secondary hyperaldosteronism. Hydrochlorothiazide use produces acquired inhibition of the Na^+^-Cl^−^ cotransporter, while Gitelman syndrome due to mutations in the gene coding the transporter causes congenital dysfunction [30, 32].

In the collecting duct, the coordinated activity of cytosolic carbonic anhydrase and both apical and basolateral ions transporters including the ENaC, H^+^-ATPases, and the kidney isoform of AE1 allow sodium recovery associated with bicarbonate reabsorption in exchange for chloride that is excreted into the urine. In addition, hydrogen ions and potassium are secreted into the tubular lumen [34–36]. Mineralocorticoids such as aldosterone stimulate sodium reabsorption in the collecting duct through activation of the ENaC and therefore allow bicarbonate recovery and chloride waste in urine [35]. Metabolic alkalosis occurs in conditions associated with excess aldosterone activity, either autonomous secretion of aldosterone (primary aldosteronism) or secondary activation of the RAA system owing to renin-producing tumors, renal artery stenosis, and intravascular volume contraction of any etiology [30, 32, 33, 52].

Metabolic alkalosis may also occur in Liddle syndrome and 11*β*-hydroxysteroid dehydrogenase deficiency, conditions that cause apparent mineralcorticoid excess. Liddle syndrome is a congenital disease caused by mutations that inhibit the removal of the ENaC from the plasma membrane, resulting in permanent sodium reabsorption via this channel. Similarly, excess cortisol levels owing to failure of inactivation of cortisol by the enzyme 11*β*-hydroxysteroid dehydrogenase activate the mineralcortidoid receptor in the collecting duct and enhance sodium reclamation by the ENaC. Deficiency of the enzyme 11*β*-hydroxysteroid dehydrogenase may be congenital or acquired due to ingestion of carbenoxolone or licorice. Both Liddle syndrome and 11*β*-hydroxysteroid dehydrogenase deficiency simulate primary hyperaldosteronism, including metabolic alkalosis, but the plasma concentrations of renin and aldosterone are low [33].

Pendred syndrome is a recessive autosomal disorder characterized by thyroid goiter and sensorineural hearing loss due to mutations in the gene that encodes pendrin, which is an apical cortical collecting duct chloride/bicarbonate exchanger that manages the secretion of bicarbonate into the tubule lumen in exchange for chloride reabsorption [37–40]. Pendred syndrome (pendrin dysfunction) may be associated with urinary chloride loss and secondary hypochloremic metabolic alkalosis, particularly during vomiting or thiazide therapy [37, 39].

### 8.4. Respiratory Alkalosis

An increase in the ventilatory rate is usually secondary to tissue hypoxia or acidosis, although primary hyperventilation occurs during voluntary forced breathing and panic disorder attacks [14, 103]. In addition, hyperventilation typically precedes the encephalopathy associated with conditions featuring hyperammonemia and normal human pregnancy is characterized by hyperventilation of unclear cause [104].

In healthy humans, voluntary hyperventilation induces urine alkalinization to prevent the progression of plasma alkalinization, as it was first reported in 1919 [105]. The reabsorption of bicarbonate and sodium ions in the kidney tubule is inhibited in response to hypocapnia, allowing the urinary excretion of sodium in excess of chloride with subsequent plasma chloride retention relative to sodium that contributes to restrain the increase in plasma pH secondary to forced breathing. The kidney compensatory response can begin within minutes and takes effect over a period of hours to days [103].

In addition to tissue acidosis, adaptive hyperventilation is physiologically induced by tissue hypoxia, which occurs upon altitude exposure. A profound alteration in plasma and urine ionic pattern takes place upon altitude exposure, intended to enhance the body capacity to uptake and transport oxygen to tissue cells and to contain the progression of plasma alkalinization.

At high altitude, the barometric pressure is progressively reduced and consequently the partial pressure of oxygen (pO_2_) in the inspired air is lower than at sea level. Sea level barometric pressure of 760 mmHg drops to 253 mmHg on the 8,848 meters summit of the Mount Everest and the pressure of oxygen in the inspired air falls from 160 mmHg at sea level to approximately 43 mmHg at the summit of Mount Everest [106].

The decrease of the pO_2_ in the inspired air induces hyperventilation immediately upon exposure to altitude in order to maintain body oxygenation. The increase in pulmonary ventilation leads to enhanced elimination of carbon dioxide by the lungs and consequently to a reduction in the arterial pCO_2_ [107–109]. In healthy subjects climbing Mount Everest breathing ambient air, the mean arterial pCO_2_ values fell with increasing altitude, while the pH values gradually increased, reaching mean values of 13.3 mmHg and 7.53 at 8,400 m, respectively [110]. The fall in the arterial pCO_2_ that occurs on acute arrival at altitude is maintained or further declined on prolonged exposure to altitude [107, 108, 111, 112]. Healthy subjects undergoing simulated exposure to altitude in artificial hypobaric chambers show similar changes than those that develop in natural high altitude sojourns [113, 114]. Normobaric hypoxia also induces a fall in the arterial pCO_2_ and a rise in the blood pH [115]. Plasma bicarbonate concentration decreases as well on acute arrival at high altitude and remains lower than at sea level during prolonged sojourns at altitude [107–109, 116]. An immediate reduction in the plasma bicarbonate concentration is also observed in healthy subjects in a hypobaric chamber simulating 3,100 m altitude [114].

Plasma chloride concentration is elevated upon acute exposure to altitude [107]. The increase in plasma chloride concentration is sustained or increased further during the entire time at high altitude [107, 108]. Plasma chloride is also elevated in healthy volunteers experiencing simulated altitude of 3,100 m in a hypobaric chamber [114]. Unlike chloride, no consistent changes in the plasma concentration of sodium have been detected upon altitude exposure in healthy humans [107, 116–120]. The increase in plasma chloride concentration with no variation in plasma sodium limits the increase in plasma pH that occurs upon high altitude exposure.

In response to altitude, the kidney modifies the urinary ionic composition, including the concentration of hydrogen ions. A rise in urine pH maintained during the duration of the exposure is observed in healthy subjects undergoing simulated altitude in hypobaric chambers [114, 121]. Similarly, the inhalation of 14% oxygen (normobaric hypoxia) induces a fall in the urinary concentration of hydrogen ions and therefore a shift of urinary pH to higher values [122]. Additionally, in response to acute exposure to high altitude, there is an increase in urinary sodium concentration [120, 123]. Simulated high altitude exposure [113, 114, 124] and normobaric hypoxia [122] elicit a similar response, enhancing sodium urinary excretion. After early acclimatization (four weeks after arrival at 5,050 m altitude) the increase in sodium urinary excretion is less evident [123].

Hyperventilation typically precedes cerebral edema and coma associated with metabolic decompensation in urea cycle disorders and other diseases characterized by hyperammonemia. Indeed, blood ammonium should be determined when consciousness deterioration is associated with respiratory alkalosis [125]. Most conditions associated with hyperammonemia and cerebral edema exhibit secondary hyperventilation, including liver failure [126], urea cycle disorders [125], ornithine transcarbamylase deficiency [127], argininosuccinate lyase deficiency [128], lysinuric protein intolerance [129], medium-chain acyl-CoA dehydrogenase MCAD deficiency [130], propionic acidemia [131], valproate administration [132], Reye's syndrome [133], urinary tract infections by urea-splitting organisms [134], and chemotherapy for the treatment of hematologic malignancy [135]. The pathogenic mechanisms that produce hyperventilation in severe hyperammonemia are unclear. Most disorders leading to acute metabolic decompensation with hyperammonemia and cerebral edema are associated with intracellular acidosis due to accumulation of different organic anions [136] and hyperventilation may be a physiological response to intracellular acidosis.

## Figures and Tables

**Table 1:** Some p*K*_*a*_ values.

	p*K*_*a*_
Phosphoric acid (H_3_PO_4_)/dihydrogen phosphate (H_2_PO_4_^−^)	1.97
Dihydrogen phosphate (H_2_PO_4_^−^)/monohydrogen phosphate (HPO_4_^2−^)	6.86
Monohydrogen phosphate (HPO_4_^2−^)/phosphate (PO_4_^3−^)	12.35
Carbonic acid (H_2_CO_3_)/bicarbonate (HCO_3_^−^)	3.77
Citric acid/citrate	3.09
Acetoacetic acid/acetoacetate	3.58
*β*-hydroxybutyric acid/*β*-hydroxybutyrate	4.39
Lactic acid/lactate	3.86
Uric acid/urate	5.75
Ammonia (NH_3_)/ammonium ion (NH_4_^+^)	9.3
